# Anxiolytics, Sedatives, and Hypnotics Prescribed by Dentists in Brazil in 2010

**DOI:** 10.1155/2017/2841549

**Published:** 2017-05-30

**Authors:** Patrícia Azevedo Lino, Maria Auxiliadora Parreiras Martins, Maria Elisa de Souza e Silva, Mauro Henrique Nogueira Guimarães de Abreu

**Affiliations:** ^1^Department of Community and Preventive Dentistry, Universidade Federal de Minas Gerais, Belo Horizonte, MG, Brazil; ^2^Department of Pharmaceutical Products, Universidade Federal de Minas Gerais, Belo Horizonte, MG, Brazil; ^3^Department of Operative Dentistry, Universidade Federal de Minas Gerais, Belo Horizonte, MG, Brazil

## Abstract

**Objective:**

To describe dental prescriptions for anxiolytics, sedatives, and hypnotics for Brazilian outpatients in 2010.

**Methods:**

A cross-sectional study was conducted using data on the use of anxiolytics, sedatives, and hypnotics from the Brazilian Health Surveillance Agency, Brazil, 2010. For each prescription, prescribed drugs and the prescribed amount were identified. Prescribed medications were classified according to Anatomical Therapeutic Chemical code. We calculated the number of Defined Daily Doses (DDD) for anxiolytics, sedatives, and hypnotics by code, their mean DDD, and DDD per inhabitant per year.

**Results:**

There were 16,436 prescriptions dispensed, including anxiolytics, sedatives, and hypnotics. These prescriptions corresponded to 3,555,780.50 mg, distributed as 2,286,200.50 mg (64.30%) of anxiolytics and 1,269,580.00 mg (35.70%) of sedatives and hypnotics. This amount allowed treating approximately 474,106 individuals (number of DDD). The anxiolytics most frequently dispensed were bromazepam (25.30%), alprazolam (19.19%), and diazepam (15.60%). Sedatives and hypnotics mostly prescribed were zolpidem (9.55%), midazolam (6.99%), and flunitrazepam (2.14%). The per capita rates (100,000 inhabitants) of anxiolytics and sedatives/hypnotics were 6.83 and 1.78, respectively.

**Conclusions:**

Benzodiazepines and derivatives were the most frequently prescribed drugs. There was a low rate of dental prescriptions for anxiolytics, sedatives, and hypnotics, although excessive doses were concentrated in the same prescription.

## 1. Introduction

Anxiety or fear of dental procedures is prevalent condition that can discourage patients from accessing dental services. Several methods are known to control these conditions, such as the use of anxiolytics, sedatives, and hypnotics. In this group, benzodiazepines are the most consumed and, when properly used, can increase the patients' well-being and the quality of dental treatment [1, 2].

In some countries, national databases on drug prescriptions of different therapeutic classes of drugs allow the evaluation of professional prescribing patterns and help promote rational drug use [3, 4]. However, knowledge about the behavior for the prescribing of and dispensing of dental anxiolytics, sedatives, and hypnotics, generated from population-based studies, is still scarce in the literature.

The prescription of anxiolytics, sedatives, and hypnotics by dentists for outpatients is legally authorized in Brazil. Drugs are dispensed mostly by private pharmacies and recorded at the National System of Management of Controlled Products (NSMCP). This is a surveillance system that collects data on health information covering production, distribution, prescription, dispensing, and consumption of drugs of interest in the field of public health. Thus, the NSMCP provides reliable information on prescription patterns of anxiolytics, sedatives, and hypnotics prescribed by dentists in Brazil [5].

This study aimed to describe dental prescriptions of anxiolytics, sedatives, and hypnotics for Brazilian outpatients in 2010.

## 2. Methods

This is a cross-sectional descriptive study on anxiolytics, sedatives, and hypnotics prescribed by dentists for Brazilian outpatients in 2010.

According to NSMCP records, the prescriptions of drugs dispensed in 2010 by all Brazilian private pharmacies were identified. The following variables were collected: prescribed drugs and the prescribed amount of each drug. Prescribed medications were classified according to the Anatomical Therapeutic Chemical (ATC) code [6]. The third, fourth, and fifth levels of the ATC classification were used to code the drugs. After that, we calculated the number of Defined Daily Doses (DDD) for anxiolytics, sedatives, and hypnotics by code, their mean of DDD, and DDD per inhabitant per year when measurement is frequently used for drugs normally prescribed for short-term use. To calculate the number of DDD, the number of dispensed units was multiplied by the total dosage (mg) contained in the drug box. The result was divided by the DDD value indicated by ATC [6, 7]. To calculate the DDD per inhabitant per year, the number of DDD per year was divided by the number of inhabitants [7].

The database was analyzed by a researcher with eight years of experience in dealing with secondary databases. A senior epidemiologist and a senior pharmacist also assessed the database to identify any inconsistencies. The data were analyzed using the software SPSS version 20.0 for Windows (IBM Corp., released in 2011, Armonk, USA). Descriptive statistical analysis was performed by calculating proportions and measurements of central tendency and variability. No confidence interval was calculated, since it was a census study.

## 3. Results

There were 16,436 prescriptions of anxiolytics, sedatives, and hypnotics dispensed by dentists. These prescriptions corresponded to the amount of 3,555,780.50 mg, divided between 2,286,200.50 mg (64.30%) of anxiolytics and 1,269,580.00 mg (35.70%) of sedatives and hypnotics. These values indicated the treatment of approximately 474,106 people, according to the number of DDD.

The anxiolytics most frequently dispensed were bromazepam (25.30%), alprazolam (19.19%), and diazepam (15.06%). The main prescribed sedatives and hypnotics were zolpidem (9.55%), midazolam (6.99%), and flunitrazepam (2.14%) (Table 1). According to the ATC, 88.70% were benzodiazepine derivatives, 10.08% were benzodiazepine-related drugs, and 1.22% were azaspirodecanedione derivatives.

The per capita rates (100,000 inhabitants) of anxiolytics and sedatives/hypnotics were 6,832 and 1,784, respectively. The mean numbers of prescribed drug boxes were 1.86 for anxiolytics and 1.51 for sedatives/hypnotics. Table 1 depicts the detailed information for each drug regarding the total mg, number of DDD, mean of DDD, DDD per inhabitant per year, number of prescribed drug boxes (mean, minimum, and maximum), amount of dispensed prescriptions, and per capita rate (100,000 inhabitants) of the dispensed drugs.

## 4. Discussion

In Brazil, anxiolytics were dispensed more often than sedatives and hypnotics in prescriptions performed by dentists, in this most recently available dataset. Benzodiazepines were the most frequently prescribed drugs. DDD values could be considered low compared to those found in other population-based studies [8, 9]. However, comparison of these indicators becomes difficult because of differences in the objective of the treatment. Medical prescription of these drugs is often chronic, while dental prescription involves short periods of time [2].

The highest amount of prescription of anxiolytics is supported by the fact that one of the main problems of dental care is anxiety, and in most cases it is not necessary to have the patient unconscious during the procedure [1, 10]. Management of anxiety in dentistry may vary according to the needs of each patient. Drug therapy in these cases is a possible approach, albeit it is not the only option and not always the most adequate [11].

Our findings show that the per capita rate (100,000 inhabitants) for dispensing drugs is very low when compared to the high prevalence of dental anxiety or dental phobia among adults, adolescents, and children. These conditions are estimated to be pertaining to approximately 9% of the adults in Brazil [12]. Dental fear and anxiety, and the need for managing dental behavior problems, may affect 9% of the child and adolescent population [13]. Therefore, the frequency of prescription of anxiolytics, sedatives, and hypnotics in our study could be considered approximately 1,000 times lower than the expected prevalence of prescriptions for patients suffering from high levels of dental anxiety who could potentially benefit from the use of those drugs. The lack of access to dental treatment could explain the low rate of dental prescription [14].

On the other hand, the average amount of prescribed anxiolytic boxes is noteworthy mainly because the use of these drugs in dentistry is mostly indicated for short periods of time. This fact suggests irrational practice in the prescription of more than one drug box of these drugs for dental purposes in Brazil. Although the use of benzodiazepines is considered to be safe for treating anxiety in dentistry, overtreatment may increase the incidence of adverse reactions, such as confusion and hallucinations, and reflect dysfunction [2].

The access to remaining units of a drug box at home may enhance the risks for the inappropriate use of the medication without professional advice (self-medication) and accidental ingestion by children and adolescents. The unit-of-use packaging is not currently available in community pharmacies in Brazil. This type of commercial presentation of a drug product would be safer and more efficient, reducing unnecessary costs, the risk for counterfeiting, and the occurrence of medication errors [15]. Thus, despite the low prescription rates for these drugs, those patients provided with drug boxes probably received an irrational prescription.

Our results suggest problems in the prescription patterns of the drugs studied. Interventions should be planned to improve access to dental services and also the quality of dental prescriptions. In Brazil, the detection of improper or abusive prescriptions requires educational or punitive actions from a Professional Board, since the Brazilian Health Surveillance Agency has no authority to enforce legislation concerning professional practice.

Limitations of this study involve the lack of dental diagnosis, since the International Classification of Diseases (ICD) was not available for prescriptions. This information is not required by Brazilian law in prescriptions of anxiolytics, sedatives, or hypnotics. Moreover, the prescriptions registered in the database of the NSMCP correspond to drugs dispensed only by private facilities. This fact would not hinder the applicability of our results because in Brazil most drugs are obtained from the private sector. Even for patients who receive public assistance (71.0% of the population), the Brazilian Public Health System* (SUS in Portuguese)* is not always prepared to offer drugs; 27.9% of Brazilian population has private insurance and the highest out-of-pocket expenditure is related to medicines. At SUS, 33.2% of patients obtained at least one of the medicines in this public service [16]. However, we do not have access to any database for evaluating such prescriptions in a national level. Studies using a secondary database might also present methodological problems related to identification and reliability of information [4]. However, to the best of our knowledge, this is the first representative study of a whole country being evaluated for the prescription patterns of anxiolytics, sedatives, and hypnotics prescribed by dentists.

Population-based measurements would help implement actions to promote the rationality of dental prescriptions and enable an effective surveillance system. More clinical and population-based investigations are needed to evaluate the rationality of the prescription of psychotropic drugs by dentists in Brazil and in other countries.

## 5. Conclusion 

Benzodiazepines and their derivatives were the most frequent subgroup of anxiolytics, sedatives, and hypnotics prescribed by dentists in Brazil. There was a low rate of dental prescriptions of these drugs, although excessive doses were concentrated in the same prescription.

## Figures and Tables

**Table 1:** Anxiolytics, sedatives, and hypnotics prescribed by dentists in Brazil in 2010.

ATC name	Total mg	Number of DDD^*∗*^	Mean of DDD^*∗∗*^	DDD per inhabitant per year	Number of drug boxes	Mean (minimum–maximum) number of boxes	Number (%) of dispensed drugs	Per capita rate (100,000 inhabitants)
Alprazolam	117,787.50	117,787.50	37.35	0.00061748	5,672	1.80 (1–12)	3,154 (19.19%)	1.653
Bromazepam	918,423.00	91,842.30	22.09	0.00048147	7,931	1.75 (1–15)	4,158 (25.30%)	2.180
Buspirone	49,100.00	1,636.67	8.14	0.00000858	347	1.73 (1–6)	201 (1.22%)	0.105
Chlordiazepoxide	2,600.00	86.67	28.89	0.00000045	7	2.33 (2-3)	3 (0.02%)	0.002
Clobazam	110,600.00	5,530.00	28.36	0,00002899	426	2.18 (1–6)	195 (1.19%)	0.102
Cloxazolam	96,270.00	NC^*∗∗∗*^	NC^*∗∗∗*^	NC^*∗∗∗*^	2,419	1.66 (1–6)	1,458 (8.87%)	0.764
Diazepam	848,550.00	84,855.00	34.27	0.00044484	4,295	1.73 (1–10)	2,476 (15.06%)	1.298
Lorazepam	142,870.00	57,148.00	41.17	0.00029959	3,133	2.26 (1–9)	1,388 (8.44%)	0.728

Total anxiolytics	2,286,200.50	358,886.13	27.54	0.00188139	24,230	1.86 (1–15)	13.033 (79.30%)	6.832

Midazolam	560,770.00	37,384.67	32.54	0.00019598	1,517	1.32 (1–15)	1,149 (6.99%)	0.602
Estazolam	4,900.00	1,633.33	24.75	0.00000856	121	1.83 (1–5)	66 (0.40%)	0.035
Flunitrazepam	20,090.00	20,090.00	57.24	0.00010532	677	1.93 (1–8)	351 (2.14%)	0.184
Flurazepam	207,900.00	6,930.00	54.14	0.00003633	231	1.80 (1–5)	128 (0.78%)	0.067
Nitrazepam	9,900.00	1,980.00	38.08	0.00001038	99	1.90 (1–3)	52 (0.32%)	0.027
Zolpidem	448,020.00	44,802.00	28.54	0.00023487	2,389	1.52 (1–9)	1570 (9.55%)	0.823
Zopiclone	18,000.00	2,400.00	27.59	0.00001258	120	1.38 (1–3)	87 (0.53%)	0.046

Total hypnotics and sedatives	1,269,580.00	115,220.00	33.86	0.00060402	5,154	1.51 (1–15)	3403 (20.70%)	1.784

^*∗*^Total mg/DDD value; ^*∗∗*^number of DDD/number of dispensed drugs; ^*∗∗∗*^NC: drug with no DDD available.
